# The Relationship between Exercise and Salivary Oxidative Stress: A Systematic Review

**DOI:** 10.3390/antiox11081489

**Published:** 2022-07-29

**Authors:** Raphael Charchar Campos Alves, Railson Oliveira Ferreira, Deborah Ribeiro Frazão, Yago Gecy de Souza Né, Paulo Fernando Santos Mendes, Guido Marañón-Vásquez, Luiz Fernando Freire Royes, Nathalia Carolina Fernandes Fagundes, Lucianne Cople Maia, Rafael Rodrigues Lima

**Affiliations:** 1 Laboratory of Functional and Structural Biology, Institute of Biological Sciences, Federal University of Pará, Augusto Corrêa Street 1, Guamá, Belém 66075-900, Brazil; raphaelcharcharalves@gmail.com (R.C.C.A.); railson_91@yahoo.com (R.O.F.); deborahfrazao@hotmail.com (D.R.F.); yagogecyne@gmail.com (Y.G.d.S.N.); paulofsmendes@gmail.com (P.F.S.M.); 2 Department of Pediatric Dentistry and Orthodontics, School of Dentistry, Federal University of Rio de Janeiro, Rio de Janeiro 21941-913, Brazil; guido_amv@hotmail.com (G.M.-V.); rorefa@terra.com.br (L.C.M.); 3 Exercise Biochemistry Laboratory, Center of Physical Education and Sports, Federal University of Santa Maria-UFSM, Santa Maria 97105-900, Brazil; nandoroyes@yahoo.com.br; 4 School of Dentistry, Faculty of Medicine and Dentistry, University of Alberta, Edmonton, AB 11405 87, Canada; nathaliacffagundes@gmail.com

**Keywords:** exercise, oxidative stress, saliva, sports

## Abstract

Salivary content has been reported as a potential biomarker for oxidative stress assessments especially in context of exercise-induced oxidative stress. This systematic review following PRISMA guidelines aimed to evaluate the effects of physical exercise and changes promoted in oxidative stress identified in saliva. Methods: Studies published up to May 2022 were searched in online databases (PubMed, Scopus, Web of Science, The Cochrane Library, LILACS, OpenGrey, and Google Scholar). Risk of bias evaluation were performed using the Quality Assessment Tool for Before-After (Pre-Post) Studies with No Control Group. Results: A total of 473 references were identified and 22 considered eligible. In this case 14 studies reported increase of antioxidant parameters in saliva while eight studies demonstrated increased lipid peroxidation after exercise. Regarding nitrite levels, two studies showed higher levels after exercise. The quality of evidence was very low due to high heterogeneity, inconsistency and indirectness among studies according Grading of Recommendations, Assessment, Development and Evaluation analysis. Conclusion: Increase of oxidative stress and antioxidant activity in saliva appears to be present after exercise, especially at moderate intensity. However, the wide variety of methods leads to divergent data. For precision in salivary assessments, new research with larger sample sizes and better participant matching are recommended.

## 1. Introduction

Physical activity and physical exercise are essential methodologies applied to improve physical capacities, prevent diseases, and strengthen social interactions with adaptations for the biopsychosocial well-being state. Both are beneficial to cardiovascular, metabolic [1], osteomyoarticular, and, recently, cognitive functions [2]. Physical exercise differs from physical activity as it considers individual planning through delimiting the volume, frequency, type of exercise, and intensity [3].

From a metabolic point of view, it is worth mentioning that the oxidative stress is a necessary and inevitable consequence of aerobic or anaerobic metabolism closely related to production of reactive oxygen species (ROS) and reactive nitrogen species (RNS) [4]. Consequently, it seems likely that cell membrane and mitochondria are both a source and a target of oxidizing agents [5]. Furthermore, the development of compensatory responses to oxidative stress induced by physical training [6,7] reflects the decrease of ROS/RNS generation markers, which translate into maintenance. This hypothesis has been supported by clinical and experimental studies, which indicate that a significant adaptive response to physical training involves a large increase in resistance capacity facilitated by increased O_2_ consumption and mitochondrial biogenesis [7,8,9]. It has been demonstrated that exercise training prevented the downregulation of relevant mitochondrial functioning parameters, such as the membrane potential (Δψ), CS (citrate synthase) and dehydrogenases activities [10]. However, despite the many benefits of exercise, we should consider that a frequent practice of high-intensity exercise may increases blood oxidative stress [7].

Considering the benefits and harmful effects of exercise-induced oxidative stress, studies report a dose-response pattern of oxidative and antioxidant parameters [11]. Pro-oxidant factors participate in the modulation of muscle strength; thus, a low level of ROS/RNS induces an adaptative effect in cell or in the organism, generating a beneficial process called hormesis by authors [7,11]. Hormesis is defined as the process in which a low dose of ROS/RNS, that is harmful in high doses, induces a beneficial adaptive control vasodilation by nitric oxide pathway, for example [12]. Even so, the possibility of oxidative stress induced by physical exercise causing damage mainly in the context of high-performance sports requires the need for methods of oxidative balance assessment that are efficient and of low cost, preferably [13].

Identifying exercise-induced oxidative stress biomarkers is still a challenge, especially in large-scale studies where the cost-effectiveness and non-invasive nature of the measures are crucial [14]. Although plasma has been the most widely utilized tissue to test reactions to physical exercise, in recent years, there are increasing evidences suggesting that saliva may also be employed as a tool to measure exercise-induced chemical changes and adaptations [15,16]. Authors reported a link between antioxidant markers in plasma and saliva in their study. Their research found that antioxidant capacity, when assessed in saliva, can be used to predict oral cavity disorders [17].

In this aspect, saliva has an essential role in the physiology of oral tissues [18]. Salivary defense functions of hard and soft tissues of the oral cavity are observed, and also includes functions of lubrication, swallowing, and digestion [19]. Furthermore, salivary content has been reported as a potential biomarker for several assessments in medical research [20] and sports [21].

## 2. Materials and Methods

### 2.1. Registration Protocol and Study Design

This systematic review was registered at Open Science Framework under the registration 10.17605/OSF.IO/ZHW9D and performed according to PRISMA (Preferred Reporting Items for Systematic Review and Meta-Analysis) guidelines [22] (Supplementary Table S1).

### 2.2. Eligibility Criteria

After developing a PICO statement, interventional studies published until March 2022 were searched in online databases. They should have been made in adult populations (P), having physical exercise as an intervention (I), pre-exercise assessment as a comparison (C), and parameters of salivary oxidative stress as an outcome (O).

Furthermore, the eligibility criteria did not restrict the year of publication and language, but only considered published manuscripts. Articles that did not follow our eligibility criteria, opinion articles, technical articles, guidelines, animal, and pilot studies were excluded.

### 2.3. Information Sources

Two reviewers (R.C.A. and R.O.F.) conducted an independent systematic search on six databases by March 2022: PubMed, Scopus, Web of Science, The Cochrane Library, LILACS, and Clinical Trials. In addition, the OpenGrey and Google Scholar were selected to perform searches on grey literature. Moreover, the reviewers conducted a hand search on the reference lists cited in the studies included in the systematic review.

### 2.4. Search Strategy

We employed a combination of pre-defined controlled MeSh (Medical Subject Headings) and free terms relating to physical exercise and oxidative stress. It was then coupled with terms “humans” or “adults” and “sports” or “athletic” or “exercise” or “athletic performance” and or “saliva” or “salivation” and “oxidative stress.” Supplementary Table S2 describes the search approach for selected databases. To execute a combination of pre-defined terms, differences in database syntax rules were examined.

### 2.5. Selection Process

Two examiners (R.C.A. and R.O.F.) conducted all searches in databases. After that, all relevant citations were saved in a bibliographic reference manager (EndNote, x9 version, Thomson Reuters). The authors subsequently deleted duplicated studies independently, maintaining only one publication. Additionally, titles and abstracts that did not meet the set eligibility criteria were rejected. The remaining articles were examined and evaluated by reading their full texts. Finally, citations were searched from the reference lists of all previously selected remaining articles throughout the full-text analysis. A third examiner (D.R.F.) was consulted if there was any disagreement.

### 2.6. Data Collection Process

The data from the research was retrieved and tabulated separately by two reviewers (R.C.C.A. and R.O.F.). When there was a controversy, another two authors were considered. The researchers consider author information, region and year of publication, study design, sample source, sample sizes, participant age, physical training evaluation, salivary oxidative stress evaluation, statistical analysis, results, and outcomes. We attempted to contact the authors through e-mail in the absence of relevant information for data extraction or risk of bias evaluation. For up to five weeks in a row, we sent the authors a weekly e-mail.

### 2.7. Quality Assessment Analysis

Assessment of the methodological quality and the risk of bias was performed using the checklist of Quality Assessment Tool for Before-After (Pre-Post) Studies with No Control Group (National Institutes of Health, Bethesda, MD). In this case, 12 questions for quality assessment involve methodological issues considering the risk of potential for selection bias, information bias, measurement bias, or confounding (the mixture of exposures that one cannot tease out from each other). Answers are “YES,” “NO,” “CD” (cannot determine), “NA” (not applicable), or “NR” (not reported) for each question regarding methods. Two reviewers (R.O.F. and D.R.F.) performed the quality rating of selected articles, and in case of disagreement, a third reviewer analyzed the quality assessment.

If the absence of information compromised data extraction or the risk of bias evaluation, an attempt to contact the authors by e-mail was made. The contact consisted of sending a weekly e-mail for five consecutive weeks.

### 2.8. Risk of Bias

Guidance for risk of bias focus on the concepts underlying the questions in the quality assessment tool. For any box checked “no,” reviewers should ask, “What is the potential risk of bias resulting from this flaw in study design or execution?” That is, does this factor lead to doubt about the results reported in the study or doubt about the ability of the study to accurately assess an association between the intervention or exposure and the outcome?

After analysis of all twelve questions, a study can be classified with a quality rate as good (low risk of bias), fair (moderate risk of bias), or poor (high risk of bias). If a study was classified as inferior, issues related to methods were described.

### 2.9. Statistical Synthesis

Because the majority of the studies selected had different methodologies and high heterogeneity, a statistical analysis of the data was not considered for this study [23]. As a result, we decided not to perform the meta-analysis in order to avoid reporting data with high heterogeneity or imprecise estimations regarding the effects of physical exercise on salivary oxidative stress [24].

### 2.10. Assessment of Quality of Evidence

The certainty of the evidence was evaluated following the GRADE approach and recommendations for when there is no single estimate of the effect. Observational studies start as moderate evidence, and the certainty of evidence decreases to low or very low quality. It happens if serious or very serious issues, related to risk of bias, inconsistency, indirectness, imprecision, and publication bias, are present. In addition, the quality of the evidence can be upgraded if the magnitude of effect is large or very large, or if the effect of all plausible confounding factors would be to reduce the effect, or suggest a spurious effect. In this way, the quality of the evidence can vary from very low to high.

## 3. Results

### 3.1. Study Selection

**Figure 1:**
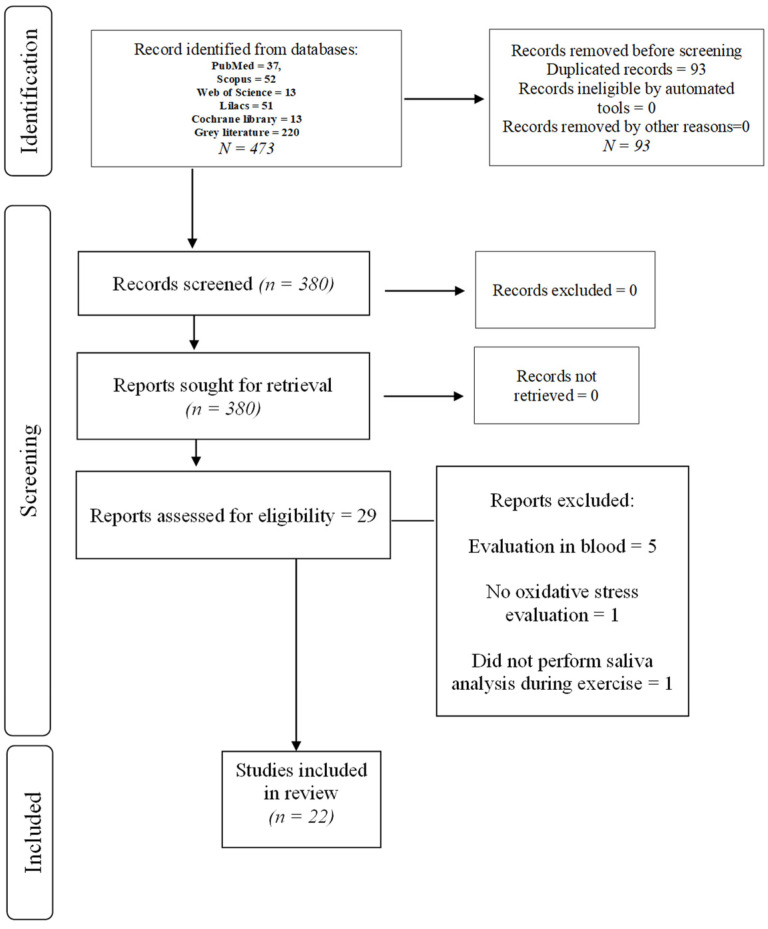
Prisma 2020 flow diagram of study selection process.

Among the 473 references identified in searching databases, 93 duplicates were removed. After reading titles and abstracts, 351 references were excluded based on the eligibility criteria; thus, 29 were selected for full-text appraisal. After that, seven studies were excluded: 5 studies conducted their oxidative stress evaluation in the blood, not in saliva [25,26,27,28,29]; one study did not perform salivary evaluations during exercise intervals [30]; and 1 article did not analyse oxidative stress parameters [31].For reasons for excluding articles, please see Supplementary Table S3. Finally, 22 articles were eligible for qualitative assessment (Figure 1).

### 3.2. Study Characteristics

Considering selected participants, the studies evaluated healthy active individuals and athletes. For example, most studies researched athletes [12,32,33,34,35,36,37] while the other articles analyzed sports practitioners. For example, judo athletes [38,39,40]; swimming and athletics [14,41,42]; weightlifting athletes [15,43]; college athletes but with no descriptions of practiced sports [44,45]; runners of a 10,000 m race [46]; soccer players [47]; military athletes of pentathlon [48]; kickboxer athletes [49]; and finally, cross-fit practitioners [50].

Salivary oxidative stress was measured at different time among studies. Eleven studies evaluated participants before and after training [15,34,38,39,40,42,43,46]. Six trials evaluated 3 periods, including one baseline assessments and two post-training assessments [32,36,37,44,45,49]. Five studies evaluated in 4 or more intervals, organized into before and after two or three training or competition sessions [12,14,35,47,50]. The saliva collection methods used by the articles were non-stimulated [14,15,32,33,34,35,36,37,38,39,40,41,42,43,44,45,48], stimulated [46] (chewing gum), and with cotton swab [12,47,50]. The spectrophotometric assay [14,15,32,33,34,35,36,37,38,41,42,43,44,45,46,47,48,49], enzymatic immunosorbent assay [12,50] (ELISA), and high-performance liquid chromatography [39,40] (HPLC) were the methods used to evaluate the parameters of oxidative stress.

Regarding the intervention, the authors evaluated many kinds of exercises, such as session of sports training [38,39,40,42,47,49]; high-intense interval exercise (HIIE) [34,36]; aerobic exercise [12,14,32,33,44,45,46]; resistance training [15,43,44,50]; acute intense exercise [35,37]; running-based anaerobic sprint test [48]; and anaerobic interval physical exercise [41]. All studies performed analysis with quasi-experimental design.

Finally, oxidative parameters were measured by the formation of products of oligosaccharides decarboxylation [38], protein oxidation [15] and lipid peroxidation, these were quantified in their initial phase by quantification dienes [39,41,42,43,49] and/or conjugated trienes [41,42] as well as for final products of lipid peroxidation by Schiff Bases [41] and isoprostane levels [14]. Additionally, malondialdehyde levels were also directly quantified [33,34,39,40,43] or indirectly by determining the levels of thiobarbituric acid reactive substances [15,34,35,47,48,49]. In addition, nitrite levels were also measured as oxidative parameters since they are increased in response to oxidative damage [12,35,36,46].

To evaluate defense mechanisms, enzymatic and non-enzymatic antioxidants were examined. Among the enzymatic antioxidants, superoxide dismutase was measured [32,34,35,36,37,38,45,49], catalase [32,34,36,37,38,43,45], glutathione peroxidase [38,40] and peroxidases [32,43]. The non-enzymatic antioxidants evaluated were reduced glutathione [15,34,36,42,43,47,48], oxidized glutathione [34,47], uric acid [15,34,35,36,45,46,47,48,50] and thiols [43,49]. In addition, they were evaluated by assessment of antioxidant capacity by the methods capturing the ABTS+ radical [33,46], radical DPPH+ [47,48] and the ability to reduce iron [35,36,44]. The summary of the characteristics and results of individual studies is shown in Table 1.

### 3.3. Results of Individual Studies and Syntheses

Ten studies evaluated lipid peroxidation parameters in which six studies reported changes in lipid peroxidation parameters. Five of them related an increase of its levels [33,41,42,48,49], while one reported a decrease [46]. Among the 5 studies that reported an increase, two of them evaluated saliva in the context of anaerobic exercise [41,42] with fair [41] and low [42] risk of bias. The other three articles evaluated in an aerobic exercise context [33,48,49] with high [33] and fair [48,49] risk of bias. The authors who reported a decrease evaluated oxidative stress after aerobic exercise [46], but the study showed high risk of bias [46]. Four studies that evaluated lipid peroxidation parameters did not show the statistical difference [15,34,40,43], with low and fair [43] risk of bias.

Besides, three studies with high [12] and low [35] risk of bias reported that Nitrite levels(NO_2_^−^) were higher after anaerobic [35] and aerobic exercise [12,36]. However, no effects on nitrite levels after aerobic exercise were reported by one study with high risk of bias [46]. Regarding antioxidant parameters, increased antioxidant enzymes were presented by 16 studies [15,32,34,35,36,37,38,39,40,41,42,45,46,47,48,49,50]. On the other hand, two articles related a reduced antioxidant capacity [33,44]. Only one reported that the total antioxidant capacity did not differ after exercise [47]. Among those antioxidant parameters, it’s worth mentioning that nine studies related an increase in uric acid levels after exercise [15,34,35,36,45,46,47,48,50].

Aerobic interventions addressed in articles were: cycle ergometer or treadmill test with increasing workloads [12,14,32,33,35,36,37,45], session of sport training [38,39,40,43,47,49], and running exercise [46]. Anaerobic interventions consisted of: hypertrophy training [15,36,44,47,50] and high-intensity intermittent exercise [34,36,41,42]. Considering the association between type of exercise and oxidative stress, an increase of antioxidant parameters was found in sports training [38,39,40,43,47,49]; aerobic exercise [32,45,46]; resistance training [15,43,50]; HIIE [34,36,42]; and acute intense exercise [35,37]. For instance, regarding resistance training, the authors found that uric acid was 47.6% higher in saliva after training (*p* < 0.01) [15]. One study reported an inverse relationship in antioxidant parameters and exercise, which the Total Antioxidant Capacity (TAC) was lower 24 h after exercise than before [44].

### 3.4. Risk of Bias

The quality of the measurements reported in each article is shown in Table 2. Only two studies had a poor quality and high risk of bias [14,46]. That happened because their eligibility criteria for the study population were not prespecified and clearly described. Also, the participants in the study were not representative enough. Besides, they did not report if the eligible participants met the prespecified entry criteria enrolled in the study, and the sample size was not sufficiently large. Nine studies presented a fair quality and medium risk of bias [12,33,38,41,43,44,45,48,49] because some of them did not clearly state the study question [12,44].

Moreover, those fair-quality studies had issues regarding representativeness and sample size. Finally, 11 articles presented a good quality and low risk of bias [15,35,36,37,39,40,42,47,50,51], with minimum issues related to blindness and sample size calculation.

Most limitations observed in studies were lacking sampling methods [12,14,15,32,33,35,36,37,38,39,40,41,42,43,44,45,46,48,49,51]. In addition, they demonstrated reduced samples with low representativeness of the population [15,38,41,43,44,45,46,48,49,51], lack of clear eligible criteria [14,15,38,41,43,45,46,48], and few descriptions of demographic characteristics for cofounding factors controlling [14,15,38,43,44,45,46]. For analysis of all checklist criteria, please see Supplementary Table S4.

### 3.5. Certainty of Evidence

In general, and without considering the type of exercise implemented or the variations in the moment of the evaluations, the certainty of the evidence was rated as very low. The studies included in the syntheses presented methodological limitations that could have seriously affected the estimates reported. For the synthesized outcomes that included more than one study, the reported results were not entirely consistent regarding the direction and size of the effects. Clearly, the methodological heterogeneity among the studies explains the inconsistency of the results. Furthermore, since uncontrolled studies were included, it cannot be affirmed that exercise is really the factor that explains the variations in the levels of the oxidative stress parameters evaluated; therefore, it was judged that item indirectness was also seriously affected. Finally, even for the outcomes that included more studies, the number of individuals considered when synthesizing the information was small; thus, it was deemed that the imprecision item was seriously affected (Table 3).

## 4. Discussion

Physical exercise appears to produce changes in salivary oxidative stress, with an increase in lipid peroxidation and nitrite levels, as well as an increase in antioxidant activity, according to the selected studies. Due to the methodological differences and limitations of the studies, such as small sample sizes, the certainty of evidence is quite low.

Selected articles of this review demonstrated that salivary content evaluation is feasible to oxidative stress assessment [51]. However, with minimization of oral diseases consequences, saliva can be used for assessment of several biomarkers of lipid peroxidation, protein oxidation, and DNA damage caused by reactive oxygen species [21]. Free radicals and reactive oxygen/nitrogen species (ROS/RNS) in saliva, such as those in plasma and tissues, play a vital role in redox-dependent signaling and are required for physiological processes [52].

Excessive generation of free radicals, on the other hand, might result in oxidative stress [53]. Thus, the redox balance is changed in favor of oxidants. ROS/RNS have the potential to cause oxidative damage to biological components, which can have major pathophysiological effects [54]. Saliva, on the other hand, contains a variety of antioxidant mechanisms, including low molecular antioxidants such as glutathione, ascorbic, and uric acid, as well as melatonin [51]. Saliva also contains antioxidant enzymes such as superoxide dismutase, catalase, and glutathione peroxidase [21,51], which protect the oral cavity from the harmful effects of both endogenous and exogenous ROS/RNS.

Lipid peroxidation is directly linked to muscular contraction during exercise due to oxidation of polyunsaturated fatty acids of cell membranes [7]. Mitochondria, phospholipase A2, and NADPH oxidases are the three main producers of superoxide radicals in muscle fibers (NOX2 and NOX4). NADPH oxidases (NOX2 and NOX4) have been identified as substantial producers of superoxide radicals in the cytosol and sarcolemma, notwithstanding the importance of mitochondrial respiratory activity. An increase in malonaldehyde and nitrite levels is often linked to short-term, high-intensity activity, whether aerobic or not [7,11].

The Thiobarbituric Acid Reactive Substances (TBARS) and Griess reactions are commonly used in assays for evaluating exercise-related lipid and protein peroxidation [43], but the Schiff reaction is also used to evaluate malondialdehyde for peroxidation [12]. In the presence of high-intensity exercise, diene and triene conjugate levels in saliva are raised, indicating probable exercise-related cellular damage. The presence of these conjugates, however, does not always imply cell injury [21]. This is due to the fact that they can be formed from processes such as muscular fatigue, lactate present, and musculoskeletal injuries [55,56]. As a result of the TBARS assay’s limited sensitivity and specificity, many contradictory results with regard to saliva were discovered in this review [35,47,48].

Authors report elevated levels of nitric oxide (NO) metabolites during and after exercise [12,36]. NO metabolites derived from reactions of 1-Arginine with Nitric Oxide Synthase isoforms (NOS) indicating protein oxidation [12,36]. Variations in NO levels are also discovered based on the intensity of training [11]. Furthermore, an increase in nitric oxide metabolites is associated with a decrease in salivary immunoglobulin A, potentially aggravating acute oral illnesses [12]. High NO salivary levels might be related to immunological reactions that possibly leads to salivary function reduction. Due to small number of studies evaluating NO metabolites, we suggest further evaluations that consider these parameters since exercise practitioners and athletes usually report hyposalivation [57].

Authors find higher blood levels of many enzymatic components such as superoxide dismutase (SOD), catalase, and peroxidase [15]; non-enzymatics such as reduced gluthatione (GSH) and uric acid; and indices such as total antioxidant capacity (TAC) when connecting exercise and antioxidant activity [7]. High levels of salivary antioxidant indices or enzymes are important to neutralize oxidative radicals in mouth [49]. In the oral cavity, an increase in SOD and catalase levels was detected, which was mostly related to the intensity of the activity, whether aerobic or anaerobic. In terms of non-enzymatic components, uric acid levels are higher in saliva, however the results are mixed among the writers. GSH, on the other hand, is shown to be enhanced in salivary levels, as measured by the TAC index [36,45,47,48]. Because antioxidant activity is dose-dependently related to physical exercise, these variations in antioxidant activity may be related to the establishment of an adaptation to the stress induced and a general condition of individual [7].

It is important to highlight that nine studies reported increased levels of uric acid associated with other antioxidant parameters. Uric acid is a by-product of purine metabolism and is related to peroxynitrite radicals scavenging [16]. In saliva, uric acid is responsible for 70–85% of salivary antioxidant status and may be related to protection of oral tissues against oxidative damage, especially when the periodontal disease, a common inflammatory of dental supporting tissues (gingiva, periodontal ligament, cement, and alveolar bone), affects individuals [16,50]. UA might also reflect systemic alterations regarding exercise since both aerobic and anaerobic exercise showed relationship with increased levels of UA. Despite the relationship between increased levels of salivary UA and exercise, only 2 of 9 studies evaluate UA and NO metabolites. Thus, further studies to evaluate UA and reduction of reactive nitrogen species are recommended.

Although exercise may be linked to changes in salivary biochemical balance, the studies reviewed have a number of flaws. The lack of control groups for matching operations is one of them. Many people object to studies without a control group since there is no way to know whether what happened to the participants would happen to a group that did not receive intervention [58]. To address some of these concerns, several researchers employ the before-and-after methodology, in which baseline data replaces data from a control group. Still, the data must be carefully evaluated, as the work with athletes and active and trained individuals, the biochemical changes of salivary oxidative stress in evaluated group may be related to internal and external validation issues [58].

Another issue that frequently arises in papers is the sample size [59]. Estimating the effects of exercise on salivary oxidative stress is easier when the number of participants is planned ahead of time based on the key outcomes [21]. Random error is common in studies with few participants, and systematic error is also typical, which is caused by difficulty in estimating a specific parameter, the influence of confounding factors, and other issues [58].

It should be highlighted that sports practice and training research presents numerous challenges [58]. The following stand out among them: Difficulties in obtaining participants who meet specific eligibility criteria, resulting in a reduction in the sample size; methods used in sports evaluation, such as VO_2_ Max, but which are impractical in certain intervention methodologies, being costly for many researchers and “time-consuming” for athletes, coaches, and investors; and finally, the compliance and adherence of athletes as well as the technical team with regard to activities outside the training routine [60]. Such problems result in many conflicting data and low clinical relevance in the context of intervention in this population. Therefore, even if there is a lot of uncertainty about the evidence of the impact of physical training and the implications of salivary oxidative stress, the data presented by the studies should be interpreted with caution.

This review has limitations. The absence of a quantitative analysis limited the conclusions about precision of data and regarding salivary oxidative stress during physical exercise [23]. However, in the context where there is a heightened of heterogeneity between studies, a meta-analysis is not recommended [24]. Another limitation of this work comprises the evaluation of articles that are observational or quasi-experimental studies. These studies lack the methodological rigor of an RCT, especially in terms of randomization and participant allocation [61]. As a result, even when executing a “pool” of data, the outcomes tend to have a reduced weight of evidence, notably when the sample size is small and with low representativeness of the population [61]. However, due to the lack of articles that present judicious methodologies, this review aims to demonstrate the methodological issues so that further studies can reduce the uncertainty of this association between exercise and salivary oxidative stress. Therefore, to increase the level of evidence about the effects of exercise on salivary oxidative stress, more randomized clinical trials or observational studies with blind evaluations, adequate matching of participants, similar measures to evaluate outcomes, and adequate control of cofounding factors are required [62].

## 5. Conclusions

In the context of physical exercise and sports practice, an increase in oxidative stress and antioxidant activity in saliva appears to be present. However, the wide variety of study methodology leads to divergent data and a low level of confidence in the evidence. For precision in saliva findings, new research with larger sample sizes and approaches that promote better participant matching are recommended.

## Figures and Tables

**Table 1:** Summary of the individual studies’ outcomes.

Author/Year/Country	Participants	Age (Mean)	Salivary Oxidative Stress Evaluation	Type of Exercise	Results	Main Conclusions
Biagini 2020 Italy	*n* = 10 swimming athletes	23 ± 5 years	Carbonyls, isoprostanes e prostanoids [5 min before the test (t0), at the maximal exercise peak (t1), 2.5 (t2), 5 min (t3) and 10 (t4) minutes after the VO_2_max] Non-stimulated collection of saliva in tubes Spectrophotometric evaluation	Incremental cycle ergometer test Workload of 25 W for the first 5 min followed by an increase of 25 W every minute until fatigue 70 rpm	**15-F2t-IsoP (pg/mL)**: t_0_: 14 ± 6; t_1_: 17 ± 7; t_2_: 12 ± 1; t_3_: 11 ± 2; t_4_: 14 ± 2;	The results showed a significant increase in the oxidative stress biomarkers (isoprostanes) during physical exercise with a marked decrease to baseline levels, 10 min after the maximum peak observed in exercise.
Cavas, 2005 Turkey	*n* = 12 males judoists	18 ± 3.2 years	FSA, SOD, CAT, GSH-Px Two intervals 2 h Pre-test 2 h Post-training Non-stimulated collection of saliva in tubes Spectrophotometric evaluation	2 h Judo training session	**SOD (IU/mg protein)**Pre-training: 1.29 ± 0.56; Post-training: 1.83 ± 0.49. **CAT (IU/mg protein)** Pre-training: 9.12 ± 1.14 Post-training: 16.27 ± 2.56; **GSH-Px (IU/mg protein)** Pre-training: 1.55 ± 0.05; Post-training: 1.80 ± 0.08. **FSA (mg protein/mL)** Pre-training: 0.182 ± 0.010; Post-training: 1.80 ± 0.020	There was an increase of all antioxidant parameters evaluated as well as an increase of FSA levels in saliva.
Damirchi, 2015 Iran	*n* = 16	24.7 ± 2.4 years	CAT, SOD, POD Pre- and post-evaluation One hour later With/without loading Non-stimulated collection of saliva in tubes Spectrophotometric evaluation	Treadmill run: Warm-up of 3 min (8.05 km/h) and gradient increase of 2.5% every 2 min until exhaustion	Increase of SOD, CAT, POD post-exercise (*p* < 0.05) with and without loading #	After exhaustive aerobic exercise, the results demonstrate a significant increase in salivary antioxidant enzymes SOD and POD, and CAT activity in response to the rise in free radicals caused by aerobic exercise.
Deminice, 2010 Brazil	*n* = 11 healthy and well-trained males	25.9 ± 2.8 years	Thiobarbituric acid reactive substances (TBARS), lipid hydroperoxides, advanced oxidation protein products (AOPP), uric acid (UA), Glutathione reduced (GSH), Pre-test/Post-test (10 min) Non-stimulated collection of saliva in tubes Spectrophotometric evaluation	Resistance hypertrophy training 3 sets of 10 reps. of bench press, cable pull down, overhead press, leg extension, leg flexion and leg press −75% 1 RM	**TBARS (µmol/L)**: Pre: 2.0 ± 1.2; Post: 2.5 ± 1.2; **Lipid hydroperoxides (µ mol H_2_O_2_ equivalents/L)**: Pre: 10.2 ± 2.6; Post: 11.4 ± 4.5; **AOPP (µmol chloramine -T equivalents/L)**: Pre: 30.8 ± 14.8; Post: 37.4 ± 17.5; **Uric acid (mg/dL)**: Pre: 2.1 ± 1.1; Post 3.1 ± 1.1 *; **GSH (mml/L)**: Pre: 0.16 ± 0.03; Post: 0.18 ± 0.01	After the resistance hypertrophy training, UA levels were significantly higher. However, the other parameters did not present a statistical difference.
Faelli, 2020 Italy	*n* = 20	CrossFit: 24.6 ± 3.4 years RT: 26.3 ± 3.6 years	Uric acid Cotton swabs and saliva collection tubes ELISA immunosorbent assay	Cross-fit and resistance training (RT) Cross-fit session: 2 min of rest between exercises 4 min of running, jumping rope; pull-ups and squats, front squats and kettlebell swings at 50–60% 1RM RT: 60 min, 3 sets of 15 reps. (i.e., bicep curls, lateral pulldowns, triceps pulldowns, bench presses, military presses, leg extensions, reverse leg curls, and seated leg presses).	**Uric acid (mg/dL)**: **CrossFitPre_1**: 8.68 ± 0.55; Post_1 11.62 ± 0.36 *; Pre_ 24: 9.18 ± 0.53; Post_24: 12.51± 0.31 * RT Pre_1: 5.42 ± 0.41; Post_1 7.18 ± 0.51 *; Pre_ 24: 6.22 ± 0.38; Post_24: 7.79 ± 0.70 *	Uric acid levels increased in both groups acutely.
Filaire 2010 France	*n* = 20 judo competitors	22.3 ± 1.4 years	GSH-Px (T1 = before the training session; T2 = after the training session; T3 = after six weeks just before the training session; T4 = after six weeks after the training session) Non-stimulated collection of saliva in tubes Fluorometric high-performance liquid chromatography–HPLC	2 h—Judo-training session Judospecific skills and drills and randori (fighting practice) with varying intensity of 85–90% of VO_2_max	**GSH-Px (U/g)**: T_1_: 48.6 ± 2.5; T_2_: 54.2 ± 23.5 *; T_3_: 45.6 ± 3.0; T_4_: 53.0 ± 2.9	A significant increase of GSH-Px was detected after the training session (*p* < 0.05).
Gonzalez, 2008 Venezuela	*n* = 24	27.21 ± 9.64 years	UA, TAC, Lipid hydroperoxides, nitrite determination (NO_2_) Stimulated collection of saliva in tubes (gum chewing) Spectrophotometric evaluation	10 km race	UA (*p* = 0.003), TAC increased (*p* < 0.0001); lipid hydroperoxides decreased (*p* < 0.0001); NO_2_ no effect (*p* > 0.05) #	Aerobic exercise-induced increased both TAC and UA.
Kontorshchikova, 2017 Russian Federation	*n* = 23 track and field athletes, swimming athletes	18.7 ± 0.6 years	DC, TC, SB Unstimulated collection of saliva in tubes Spectrophotometric evaluation	Anaerobic interval physical exercise 3×100 m distances by a flat race with an active 45 s rest between them for the track and field athletes, 4 × 50 m by the main swimming style with an active rest between the distances also for 45 s for the swimmers.	**DC (relative units)**: Before exercise: 0.29 ± 0.01; After exercise: 0.31 ± 0.02. **TC (relative units)**: Before exercise: 0.35 ± 0.03; After exercise: 0.49 ± 0.04. **SB (relative units)**: Before exercise: 99.94 ± 9.41; After exercise: 189.12 ± 7.69. **SB/(DC + TC) (relative units)**: Before exercise 158.65 ± 9.22; After exercise 237.88 ± 8.84.	There was an increase of lipid peroxidation levels (DC, TC, SB) after physical exercise.
Mahdivand, 2013 Iran	*n* = 20 athlete students	23 ± 2 years	TAC Non-stimulated collection of saliva in tubes ELISA	Session training concurrent (aerobic and resistance) for 100 min. 10 min warm-up (moderate running and stretching) 20 min of running with 85–80% of maximum heart rate (MHR); 10 min of active recovery; followed by 50 min (opening leg, chest press, back thigh, underarm stretch, triceps and biceps) in three sets of 6–8 repetitions at 85–90% of 1RM; cooling step to 10 min (walking and stretching)	**Total antioxidant capacity (µL. mL^−1^)**: Before: 1.96 ± 0.199; 1 h After: 1.74 ± 0.222; 24 h After: 1.78 ± 0.217.	Training concurrent (aerobic—resistance) can significantly reduce salivary total antioxidant levels.
Massart, 2012 France	*n* = 28 female judoists	23.4 ± 1.8 years	MDA, Lipid peroxides (POOL), GPx T1 Pre-test/T2 Post-test 20 min/10 min Non-stimulated collection of saliva in tubes Fluorometric high-performance liquid chromatography—HPLC	Judo training session	**Cdmax (UA)**: NCU: T_1_: 93.4 ± 9.5; T_2_: 125.4 ± 11.3; **Rmax (UA): NCU**: T_1_: 1.33 ± 0.2; T_2_: 1.22 ± 0.1; **MDA (µg.Ml^−1^)**: **NCU**: T1: 0.04 ± 0.01; T2: 0.07 ± 0.01; **POOL: NCU**: T1: 278.9 ± 24.6; T2: 378.0 ± 13.9; **GPx (U.g^−1^) NCU**: T1: 62.5 ± 4.3; T2: 73.1 ± 2.3.	Training was able to increase the levels of some antioxidants in athletes.
Menezes, 2019 Brazil	*n* = 14	22 ± 1 years	NO_2_^–^, Alpha-Amylase, Lactate, UA, TAC, TBARS, SOD. Non-stimulated collection of saliva in tubes Spectrophotometric evaluation	acute intense exercise Cycle ergometer 35-watt increments every 2 min and a fixed rotation of 70 rpm until exhaustion At least 30 min of test	NO decrease (*p* < 0.05) UA increased (*p* < 0.05) TBARS decrease (*p* < 0.05) #	There was an increased salivary level of NO, uric acid and total antioxidant capacity (TAC), reduced superoxide dismutase (SOD) activity and TBARS levels.
Nobari, 2021 Iran	*n* = 40 young men	22.93 ± 1.76 years	POX, SOD, CAT Non-stimulated collection of saliva in tubes Spectrophotometric evaluation	Pre- and post-acute intense exercise, and after one hour Treadmill run 8.05 km/h for three minutes. After 3 min the incline was set to 2.5% for every 2 min	POX—F (1, 263.49). *p* < 001 CAT—F (2135.79). *p* < 0.001 SOD—F(1.33,108.02) *p* < 0.001 #	The results demonstrate that intense and acute exercise increases the antioxidant capacity even after one hour after training.
Ovchinnikov, 2019 Russia	*n* = 70; cyclical sports (swimming and athletics)	16–20 Years	DC, TC, GSH Non-stimulated collection of saliva in tubes Spectrophotometric evaluation	Running and swimming training 3 × 100 m distances by a flat race with an active 45 s rest between them for the track and field athletes, 4 × 50 m by the main swimming style with an active rest between the distances also for 45 s for the swimmers.	**Swimmers (*n* = 40)** **DC Before**: 0.28 ± 0.001 **After**: 0.28 ± 0.002 **TC** **Before**: 0.35 ± 0.004 **After**: 0.37 ± 0.004 * GSH **Before**: 127.23 ± 3.42 **After**: 147.24 ± 4.81 * General athletes (*n* = 30) **DC** **Before**: 0.28 ± 0.003 **After**: 0.29 ± 0.004 * TC **Before**: 0.33 ± 0.009 **After**: 0.34 ± 0.008* GSH **Before**: 89.27 ± 3.59 **After**: 141.83 ± 7.50 *	Physical activities with maximum power with rest intervals stimulate the generation of MDA and increased levels of GSH for track and field and swim athletes.
Podrigalo, 2015 Ukraine	*n* = 26 weightlifting athletes	22.13 ± 3.24 years	DC, TC, (TBARS) CAT, SH, GSH Non-stimulated collection of saliva in tubes Spectrophotometric evaluation	Group 1- wrestling’s competition 1a: experienced sportsmen; 1b: beginners’ sportsmen group 2- training Competition and dynamic training loads	Malonic dialdehyde (µmol/l): **1a group**: Before: 6.86 ± 1.96; After: 4.77 ± 1.03. **1b group**: Before: 3.08 ± 0.69, After: 6.85 ± 1.14 *. **2 group**: Before: 3.12 ± 0.35; After: 8.18 ± 1.60 * **Diene conjugates (µmol/L)**: **1a group**: Before: 41.26 ± 4.78; After: 29.55 ± 3.21 *. **1b group**: Before: 27.88 ± 2.87, After: 38.54 ± 3.50 *. **2 group**: Before: 28.38 ± 1.11; After: 83.33 ± 9.69 *. **CAT (µcat/L)** **1a group**: Before: 4.28 ± 0.57; After: 4.19 ± 0.39. **1b group**: Before: 2.63 ± 0.35, After: 4.98 ± 0.47 *. **2 group**: Before: 1.78 ± 0.21; After: 3.91 ± 0.51 **GSH (mmol/L)**: **1a group**: Before: 3.18 ± 0.66; After: 3.64 ± 0.57. **1b group**: Before: 2.82 ± 0.51, After: 3.45 ± 0.45. **2 group**: Before: 1.39 ± 0.20; After: 3.10 ± 0.35 * SH-groups (mmol/L) **1a group**: Before: 2.26 ± 0.59; After: 1.68 ± 0.47. **1b group**: Before: 1.31 ± 0.34, After: 1.93 ± 0.23. **2nd group**: Before: 1.05 ± 0.14; After: 2.36 ± 0.39 *	Bio-chemical criteria of different skillfulness sportsmen illustrate different degree of stability and capacity of adaptation potentials.
Rodrigues de Araujo, 2018 Brazil	*n* = 32	21.2 ± 4.2 years	SOD, CAT, GSH, GSSG, TBARS, MDA, Uric Acid Non-stimulated collection of saliva in tubes Spectrophotometric evaluation	high-intensity interval exercise (HIIE) Successive 40 m sprints with direction changes Bangsbo sprint test	**TBARs (nmol/mL)**: Pre: 9.20 ± 3.13; Post: 8.50 ± 2.43; **MDA (µM)**: Pre: 5.40 ± 2.15; Post: 5.37 ± 1.52; **GSSG (µM)**: Pre: 2.04 ± 1.18; Post: 2.10 ± 1.13; **Uric acid (mg/dL)**: Pre: 2.66 ± 1.33; Post: 1.66 ± 0.92 *; **SOD (U/g.dL^−1^)**: Pre: 32.59 ± 43.88; Post: 37.41 ± 42.05; **CAT (U/g.dL^−1^)**: Pre: 1.65 ± 1.53; Post: 1.66 ± 2.90.	In terms of redox homeostasis, the authors saw varying findings for TBARs, MDA, GSH, GSSG, CAT, and SOD, while uric acid decreased significantly.
Sant’Anna, 2016 Brazil	*n* = 7 military athletes	27.1 ± 5.4 years	TBARS, TAC, GSH, UA Non-stimulated collection of saliva in tubes Spectrophotometric evaluation	Exercise test (RAST) warm up-5 min + 6 × 35 m sprint.	**TBARS (µM)**: Pre: 0.9 ± 0.2; Post: 1.9 ± 0.2. **TAC**: It increased by 46.6% after exercise compared to before. **GSH**: There was no significant change # **Uric acid (µM)**: Pre 178.9 ± 21.4; Post: 293.5 ± 9.4	RAST triggers free radical production, as evaluated by lipid peroxidation in saliva, and at the same time reveals an increased antioxidant activity as a adaption.
Sariri, 2013 Iran	*n* = 28 male athlete university students	22.9 ± 1.5 years	UA, CAT, POX, SOD Non-stimulated collection of saliva in tubes Spectrophotometric evaluation	Treadmill Run Astrand test at 8.01 km/h	Both enzymatic and non-enzymatic antioxidants increased immediately significantly after exercise. #	Aerobic exercise until exhaustion increases the activity of SOD, catalase, peroxidase in saliva of athlete men.
Sari-Sarraf, 2016 Iran	*n* = 27	18–21 years	MDA, TAC Non-stimulated collection of saliva in tubes Spectrophotometric evaluation	a progressive exercise to exhaustion on treadmill	**TAC (µmol/mL)**: Pratice: Pre: 0.79 ± 0.20; Post: 0.88 ± 0.17; Exautive: 0.87 ± 0.21; Control: Pre: 0.77 ± 0.18; Post: 0.79 ± 0.23; Exautive: 0.77 ± 0.23; **MDA (nmol/mL)**: Pratice: Pre: 0.48 ± 0.14; Post: 0.51 ± 0.17; Exautive: 0.54 ± 0.16; Control: Pre: 0.52 ± 0.17; Post: 0.49 ± 0.23; Exautive: 0.46 ± 0.10	Physical activity promoted an increase in lipid peroxidation and reduced antioxidant capacity, additionally it was observed that the increase in lipid peroxidation was lower in the trained group, demonstrating that physical conditioning can induce a protective effect against lipid peroxidation
Sone, 2019 Japan	*n* = 9 healthy men	23.8 ± 1.4 years	NO levels Cotton swabs and saliva collection tubes ELISA immunosorbent assay	Cycling After a 10-min warm-up, subjects cycled for 50 min at 80% VO2peak	**Nitrite (µmol/L)**: Pre-exercise: Pre: 447 ± 65; Post 0 h: 353 ± 57; Post 1 h: 367 ± 56; Post 2 h: 355 ± 49; Post 3 h: 303 ± 44. Exercise: Pre: 388 ± 82; Post 0 h: 380 ± 76; Post 1 h: 389 ± 66; Post 2 h: 401 ± 63; Post 3 h: 365 ± 53.	The results demonstrate that salivary NO levels are increased because of exercise-related stress.
Souza, 2019 Brazil	*n* = 13 healthy men	27.62 ± 1.28 years	NO, TAC, SOD, CAT, GSH, UA Non-stimulated collection of saliva in tubes Spectrophotometric evaluation	Resistance exercise (RE): 3 sets of 12-repetition maximum (12- RM) in squat (smith machine), leg press 45°, lying leg curl, and stiff exercises, in that order, with a 2 min recovery interval between sets and exercises. High-Intensity Interval Exercise (HIIE): 1 min cycling bouts at 100% of wVO2max, interspersed with 1 min of passive recovery periods at 40% of VO2max until voluntary exhaustion. The continuous exercise (CE) protocol: continuous cycling for 60 min at 50–60% of wVO2max.	NO decrease (*p* < 0.05) -RT, HIIE, and CT TAC, SOD, CAT, GSH, UA increased (*p* < 0.05) for HIIE and CE #	The results demonstrate an increase in the activity levels of amylase, total protein, and salivary nitric oxide. Additionally, in RE showed a small increase in antioxidants, while in HIIE and CE this response was more accentuated.
Viana-gomes, 2018 Brazil	*n* = 8 soccer players	27.2 ± 5.5 years	TAC, TBARS, UA. Cotton swabs and saliva collection tubes Spectrophotometric evaluation	48 h post-game-one (day 4): 1-h training session designed to simulate a game 72 h Post-game one (day 5): Resistance training consisted of 3 sets of leg press, leg extensions, leg curls, power cleans and calf raises each with self-suggested recovery intervals between sets and exercises. 24 h post-game two (day 7): low-intensity jogging for15 min (~60% heart rate peak) and low-intensity (i.e., 2 sets per exercise) resistance training over 30 min.	**UA (IU/dL)** Basal: 2.5 ± 0.3; P-G1: 2.2 ± 0.4; 48 P-G1: 2.5 ± 2.1; P-G2: 2.4 ± 0.8; 24 P-G2: 2.6 ± 2.5; 48 P-G2: 1.5 ± 0.2. **TBARS (µmol/L)** Basal: 2.1 ± 0.3; P-G1: 3.1 ± 0.4; 48 P-G1: 2.0 ± 0.2; P-G2: 3.4 ± 0.4; 24 P-G2: 2.2 ± 0.3; 48 P-G2: 2.3 ± 0.2.	The results showed an increase in TBARS after both games compared to uric acid reduction after 48 h. The antioxidant capacity did not differ.
Volodchenko, 2019 Ukraine	*n* = 18 kickboxers	17.29 ± 0.31 years	MDA TBARS, CAT, SOD, Sh-group concentration Non-stimulated collection of saliva in tubes Spectrophotometric evaluation	Training session of 110–130 min warm-up, general development exercises for all muscle groups and exercises stretching (30–35 min); main session block, kicking and striking techniques (40–45 min) and sparring (30–35 min); cool down, breathing and relaxation exercises (10–15 min).	**MDA (µmol/L)**: Before: 4.57 ± 0.25; After: 9.81 ± 0.25 *; **Diene conjugates (µmol/L)**: Before: 24.46 ± 0.31; After: 37.79 ± 0.53 *; **CAT (µKat/L)**: Before: 41.71 ± 0.35; After: 47.85 ± 0.79 *; **SH-groups (µmol/L)**: Before: 2.08 ± 0.16; After: 0.85 ± 0.13 *; **SOD** Before: 2.07 ± 0.17; After:3.48 ± 0.24 *;	Increased levels of MDA, DC, SH were found after training session. Antioxidant parameters were also reported with elevated levels.

* Statistical significance (*p* < 0.05); # The study did not include the numerical values (e.g., Mean, median, standard deviation, etc.); CAT—Catalase; DC—Diene conjugates; FSA—Free Sialic Acid; GSH—Reduced Gluthatione; GSH-Px—Reduced Gluthatione and peroxidase activity; MDA—Malonaldehyde; NO—Nitric Oxide; POX—Peroxidase; SH—Sulfhydryls groups; SOD—Superoxide dismutase; TBARS—Thiobarbituric acid reactive substances; TAC—Total antioxidant capacity; TC—Triene conjugates; UA—Uric acid; GSSG—Oxidized gluthatione; 1 RM—One repetition maximum; RT—Resistance training.

**Table 2:** Quality Assessment Tool for Before-After (Pre-Post) Studies with No Control Group.

Risk of bias	Biagini, 2020	Damirchi, 2015	Faelli, 2020	Filaire. 2010	Massart, 2012	Menezes, 2019	Nobari, 2021	Ovchinnikov, 2019	Sari-Sarraf, 2016	Sone, 2019	Souza, 2019	Viana-Gomes, 2018
Quality Rating	POOR	GOOD	GOOD	GOOD	GOOD	GOOD	GOOD	GOOD	POOR	POOR	GOOD	GOOD
**Risk of bias**	**Cavas, 2005**	**Gonzalez, 2008**	**Deminice, 2010**		**Mahdivand, 2013**	**Sariri, 2013**	**Podrigalo, 2015**	**Sant’anna, 2016**	**Kontorschikova, 2017**	**De Araújo, 2018**		**Volodchenko, 2019**
Quality Rating	FAIR	POOR	GOOD		FAIR	FAIR	FAIR	FAIR	FAIR	GOOD		FAIR

**Table 3:** Summary of findings and level of evidence following GRADE guideline.

Certainty Assessment							Impact	Certainty	Importance
№ of Studies	Study Design	Risk of Bias	Inconsistency	Indirectness	Imprecision	Other Considerations			
Lipid peroxidation after exercise									
8	observational studies	not serious	serious ^a^	not serious	serious ^b^	all plausible residual confounding would reduce the demonstrated effect	Lipid peroxidation was evaluated by different methods (TBARS, Diene and triene conjugates, isoprostanes). Six studies showed increased levels of lipid peroxidation after sessions of exercise while two studies did not. Limitations regarding sample size may affected results with no differences.	⨁◯◯◯Very low	IMPORTANT
Nitrite levels after exercise									
2	observational studies	not serious	serious ^c^	not serious	serious ^d^	all plausible residual confounding would reduce the demonstrated effect	Higher nitrite levels in saliva were found in studies. However, absence of other evaluations compromised global analysis of oxidative balance. Exercise effects related to nitrite levels may suffer alterations by cofounding factors	⨁◯◯◯Very low	IMPORTANT
Antioxidant parameters after exercise									
14	observational studies	not serious	serious ^e^	not serious	serious ^f^	all plausible residual confounding would reduce the demonstrated effect	Evaluation of antioxidant parameters was the predominant analysis. Different methods were employed (GSH, TAC, CAT, SOD) leading to high heterogeneity. Some authors did not performed global analysis of oxidant parameters and difficult to assess real effects of exercise. Reduced sample sizes and absence of cofounding factors also compromised oxidative stress evaluation in salivary content	⨁◯◯◯Very low	IMPORTANT

Explanations ^a^. High heterogeneity among lipid peroxidation parameters ^b^. Sample sizes did not reach optimal information size ^c^. High heterogeneity regarding nitrite levels assessments ^d^. Reduced sample sizes to estimate real effect ^e^. Different evaluation methods between studies ^f^. Estimation of the effect compromised due to small sample sizes.

## Supplementary Materials

The following supporting information can be downloaded at: https://www.mdpi.com/article/10.3390/antiox11081489/s1, Table S1: Prisma 2020 checklist; Table S2: Terms used on databases searches. Table S3. Articles excluded after reading in full and reasons for exclusions; Table S4: Quality Assessment Tool for Before-After (Pre-Post) Studies with No Control Group.
