# Supplementary material for: The Relationship between Exercise and Salivary Oxidative Stress: A Systematic Review
Source: Antioxidants (Basel). 2022 Jul 29;11(8):1489. doi: 10.3390/antiox11081489 (PMC9405445; doi:10.3390/antiox11081489)
Supplement: Supplementary file 1 [file antioxidants-11-01489-s001.zip › antioxidants-1801661-supplementary.pdf]

Supplementary Material

Table S1. Prisma 2020 checklist.

Section and Topic	Item #	Checklist item	Location where item is reported
**TITLE**			
Title	1	Identify the report as a systematic review.	Line 1-2
**ABSTRACT**			
Abstract	2	See the PRISMA 2020 for Abstracts checklist.	Line 17-31
**INTRODUCTION**			
Rationale	3	Describe the rationale for the review in the context of existing knowledge.	Line 35-64
Objectives	4	Provide an explicit statement of the objective(s) or question(s) the review addresses.	Line 65-71
**METHODS**			
Eligibility criteria	5	Specify the inclusion and exclusion criteria for the review and how studies were grouped for the syntheses.	Line 78-86
Information sources	6	Specify all databases, registers, websites, organisations, reference lists and other sources searched or consulted to identify studies. Specify the date when each source was last searched or consulted.	Line 88-92
Search strategy	7	Present the full search strategies for all databases, registers and websites, including any filters and limits used.	Table S2
Selection process	8	Specify the methods used to decide whether a study met the inclusion criteria of the review, including how many reviewers screened each record and each report retrieved, whether they worked independently, and if applicable, details of automation tools used in the process.	Line 88-99
Data collection process	9	Specify the methods used to collect data from reports, including how many reviewers collected data from each report, whether they worked independently, any processes for obtaining or confirming data from study investigators, and if applicable, details of automation tools used in the process.	Line 101-108;
Data items	10a	List and define all outcomes for which data were sought. Specify whether all results that were compatible with each outcome domain in each study were sought (e.g. for all measures, time points, analyses), and if not, the methods used to decide which results to collect.	Line 110-117
	10b	List and define all other variables for which data were sought (e.g. participant and intervention characteristics, funding sources). Describe any assumptions made about any missing or unclear information.	Line 110-117
Study risk of bias assessment	11	Specify the methods used to assess risk of bias in the included studies, including details of the tool(s) used, how many reviewers assessed each study and whether they worked independently, and if applicable, details of automation tools used in the process.	Line 119-130
Effect measures	12	Specify for each outcome the effect measure(s) (e.g. risk ratio, mean difference) used in the synthesis or presentation of results.	Line 142-146
Synthesis methods	13a	Describe the processes used to decide which studies were eligible for each synthesis (e.g. tabulating the study intervention characteristics and comparing against the planned groups for each synthesis (item #5)).	Line 148-156
	13b	Describe any methods required to prepare the data for presentation or synthesis, such as handling of missing summary statistics, or	Line 142-146

Section and Topic	Item #	Checklist item	Location where item is reported
		data conversions.	
	13c	Describe any methods used to tabulate or visually display results of individual studies and syntheses.	Line 148-156
	13d	Describe any methods used to synthesize results and provide a rationale for the choice(s). If meta-analysis was performed, describe the model(s), method(s) to identify the presence and extent of statistical heterogeneity, and software package(s) used.	Line 148-156
	13e	Describe any methods used to explore possible causes of heterogeneity among study results (e.g. subgroup analysis, meta-regression).	Line 142-146
	13f	Describe any sensitivity analyses conducted to assess robustness of the synthesized results.	---
Reporting bias assessment	14	Describe any methods used to assess risk of bias due to missing results in a synthesis (arising from reporting biases).	Line 148-156
Certainty assessment	15	Describe any methods used to assess certainty (or confidence) in the body of evidence for an outcome.	Line 148-156
**RESULTS**			
Study selection	16a	Describe the results of the search and selection process, from the number of records identified in the search to the number of studies included in the review, ideally using a flow diagram.	Line 159-165<br>Figure 1
	16b	Cite studies that might appear to meet the inclusion criteria, but which were excluded, and explain why they were excluded.	Table S3
Study characteristics	17	Cite each included study and present its characteristics.	Line 169-213; Table 1
Risk of bias in studies	18	Present assessments of risk of bias for each included study.	Table 2; Line 241-256
Results of individual studies	19	For all outcomes, present, for each study: (a) summary statistics for each group (where appropriate) and (b) an effect estimate and its precision (e.g. confidence/credible interval), ideally using structured tables or plots.	Line 217-234<br>Table 1
Results of syntheses	20a	For each synthesis, briefly summarise the characteristics and risk of bias among contributing studies.	Line 217-234
	20b	Present results of all statistical syntheses conducted. If meta-analysis was done, present for each the summary estimate and its precision (e.g. confidence/credible interval) and measures of statistical heterogeneity. If comparing groups, describe the direction of the effect.	--
	20c	Present results of all investigations of possible causes of heterogeneity among study results.	Line 256-268
	20d	Present results of all sensitivity analyses conducted to assess the robustness of the synthesized results.	--
Reporting biases	21	Present assessments of risk of bias due to missing results (arising from reporting biases) for each synthesis assessed.	--
Certainty of evidence	22	Present assessments of certainty (or confidence) in the body of evidence for each outcome assessed.	Line 256-268
**DISCUSSION**			
Discussion	23a	Provide a general interpretation of the results in the context of other evidence.	Line 279-282
	23b	Discuss any limitations of the evidence included in the review.	Line 364-374
	23c	Discuss any limitations of the review processes used.	Line 364-374
	23d	Discuss implications of the results for practice, policy, and future	Line 352-363

Section and Topic	Item #	Checklist item	Location where item is reported
		research.	
**OTHER INFORMATION**			
Registration and protocol	24a	Provide registration information for the review, including register name and registration number, or state that the review was not registered.	Line 74-77
	24b	Indicate where the review protocol can be accessed, or state that a protocol was not prepared.	Line 74-77
	24c	Describe and explain any amendments to information provided at registration or in the protocol.	Line 74-77
Support	25	Describe sources of financial or non-financial support for the review, and the role of the funders or sponsors in the review.	Line 389-391; 397-399
Competing interests	26	Declare any competing interests of review authors.	Line 397-399
Availability of data, code and other materials	27	Report which of the following are publicly available and where they can be found: template data collection forms; data extracted from included studies; data used for all analyses; analytic code; any other materials used in the review.	Supplementary file 2 and 3

Table S2. Terms used on databases searches.

Database	**Search format**
**PUBMED** **N=37**	((((((Saliva[MeSH Terms]) OR Saliva[Title/Abstract]) OR Salivas[Title/Abstract])) AND (((((Oxidative Stress[MeSH Terms]) OR Oxidative Stress[Title/Abstract]) OR Oxidative Stresses[Title/Abstract]) OR Stresses, Oxidative[Title/Abstract]) OR Stress, Oxidative[Title/Abstract])) AND (((((((Humans[MeSH Terms]) OR Humans[Title/Abstract]) OR Man, Modern[Title/Abstract]) OR Modern Man[Title/Abstract]) OR Man Taxonomy[Title/Abstract]) OR Homo sapiens[Title/Abstract]) OR Human[Title/Abstract])) AND ((Sports[MeSH Terms]) OR Sports[Title/Abstract]) OR Sport[Title/Abstract]) OR Athletics[Title/Abstract]) OR Athletic[Title/Abstract]) OR Athletic Performance[MeSH Terms]) OR Athletic Performance[Title/Abstract]) OR Athletic Performances[Title/Abstract]) OR Performance, Athletic[Title/Abstract]) OR Performances, Athletic[Title/Abstract]) OR Sports Performance[Title/Abstract]) OR Performance, Sports[Title/Abstract]) OR Performances, Sports[Title/Abstract]) OR Sports Performances[Title/Abstract]) OR Exercise[MeSH Terms]) OR Exercise[Title/Abstract]) OR Exercises[Title/Abstract]) OR Physical Activity[Title/Abstract]) OR Activities, Physical[Title/Abstract]) OR Activity, Physical[Title/Abstract]) OR Physical Activities[Title/Abstract]) OR Exercise, Physical[Title/Abstract]) OR Exercises, Physical[Title/Abstract]) OR Physical Exercise[Title/Abstract]) OR Physical Exercises[Title/Abstract]) OR Acute Exercise[Title/Abstract]) OR Acute Exercises[Title/Abstract]) OR Exercise, Acute[Title/Abstract]) OR Exercises, Acute[Title/Abstract]) OR Exercise, Isometric[Title/Abstract]) OR Exercises, Isometric[Title/Abstract]) OR Isometric Exercises[Title/Abstract]) OR Isometric Exercise[Title/Abstract]) OR Exercise, Aerobic[Title/Abstract]) OR Aerobic Exercise[Title/Abstract]) OR Aerobic Exercises[Title/Abstract]) OR Exercises, Aerobic[Title/Abstract]) OR Exercise Training[Title/Abstract]) OR Exercise Trainings[Title/Abstract]) OR Training, Exercise[Title/Abstract]) OR Trainings, Exercise[Title/Abstract]) OR Physical Fitness[MeSH Terms]) OR Physical Fitness[Title/Abstract]) OR Fitness, Physical[Title/Abstract])

**SCOPUS**<br><br>**N=52**	((TITLE-ABS-KEY(Humans) OR TITLE-ABS-KEY("Man, Modern") OR TITLE-ABS-KEY("Modern Man") OR TITLE-ABS-KEY("Man Taxonomy") OR TITLE-ABS-KEY("Homo sapiens") OR TITLE-ABS-KEY(Human))) AND ((TITLE-ABS-KEY(Sports) OR TITLE-ABS-KEY(Sport) OR TITLE-ABS-KEY(Athletics) OR TITLE-ABS-KEY(Athletic) OR TITLE-ABS-KEY("Athletic Performance") OR TITLE-ABS-KEY("Athletic Performances") OR TITLE-ABS-KEY("Sports Performance") OR TITLE-ABS-KEY("Sports Performances") OR TITLE-ABS-KEY(Exercise) OR TITLE-ABS-KEY(Exercises) OR TITLE-ABS-KEY("Physical Activity") OR TITLE-ABS-KEY("Physical Activities") OR TITLE-ABS-KEY("Physical Exercise") OR TITLE-ABS-KEY("Physical Exercises") OR TITLE-ABS-KEY("Acute Exercise") OR TITLE-ABS-KEY("Acute Exercises") OR TITLE-ABS-KEY("Isometric Exercises") OR TITLE-ABS-KEY("Isometric Exercise") OR TITLE-ABS-KEY("Aerobic Exercise") OR TITLE-ABS-KEY("Aerobic Exercises") OR TITLE-ABS-KEY("Exercise Training") OR TITLE-ABS-KEY("Exercise Trainings") OR TITLE-ABS-KEY("Physical Fitness"))) AND ((TITLE-ABS-KEY("Oxidative Stress") OR TITLE-ABS-KEY("Oxidative Stresses"))) AND ((TITLE-ABS-KEY(Saliva) OR TITLE-ABS-KEY(Salivas)))
**COCHRANE**<br><br>**N=13**	humans OR "Man, Modern" OR "Modern Man" OR "Man Taxonomy" OR "Homo sapiens" OR human in Title Abstract Keyword AND Sports OR Sport OR Athletics OR Athletic OR "Athletic Performance" OR "Athletic Performance" OR "Athletic Performances" OR "Performance, Athletic" OR "Performances, Athletic" OR "Sports Performance" OR "Performance, Sports" OR "Performances, Sports" OR "Sports Performances" OR Exercise OR Exercise OR Exercises OR "Physical Activity" OR "Activities, Physical" OR "Activity, Physical" OR "Physical Activities" OR "Exercise, Physical" OR "Exercises, Physical" OR "Physical Exercise" OR "Physical Exercises" OR "Acute Exercise" OR "Acute Exercises" OR "Exercise, Acute" OR "Exercises, Acute" OR "Exercise, Isometric" OR "Exercises, Isometric" OR "Isometric Exercises" OR "Isometric Exercise" OR "Exercise, Aerobic" OR "Aerobic Exercise" OR "Aerobic Exercises" OR "Exercises, Aerobic" OR "Exercise Training" OR "Exercise Trainings" OR "Training, Exercise" OR "Trainings, Exercises" OR "Physical Fitness" OR "Physical Fitness" OR "Fitness, Physical" in Title Abstract Keyword AND "Oxidative Stress" OR "Oxidative Stress" OR "Oxidative Stresses" OR "Stresses, Oxidative" OR "Stress, Oxidative" in Title Abstract Keyword AND Saliva OR Salivas in Title Abstract Keyword - (Word variations have been searched)
**WEB OF SCIENCE**<br><br>**N=13**	TS=(Humans OR "Man, Modern" OR "Modern Man" OR "Man Taxonomy" OR "Homo sapiens" OR Human) AND TS=(Sports OR Sport OR Athletics OR Athletic OR "Athletic Performance" OR "Athletic Performances" OR "Performance, Athletic" OR "Performances, Athletic" OR "Sports Performance" OR "Performance, Sports" OR "Performances, Sports" OR "Sports Performances" OR Exercise OR Exercises OR "Physical Activity" OR "Activities, Physical" OR "Physical Activities" OR "Activity, Physical" OR "Exercise, Physical" OR "Exercises, Physical" OR "Physical Exercise" OR "Physical Exercises" OR "Acute Exercise" OR "Acute Exercises" OR "Exercise, Acute" OR "Exercises, Acute" OR "Exercise, Isometric" OR "Exercises, Isometric" OR "Isometric Exercises" OR "Isometric Exercise" OR "Exercise, Aerobic" OR "Aerobic Exercises" OR "Exercises, Aerobic" OR "Exercise Training" OR "Exercise Trainings" OR "Training, Exercise" OR "Trainings, Exercise" OR "Physical Fitness" OR "Fitness, Physical") AND TS=("Oxidative Stress" OR "Oxidative Stresses" OR "Stresses, Oxidative" OR "Stress, Oxidative") AND TS=(Saliva OR Salivas)
**LILACS**<br><br>**N=51**	tw:((tw:(humans OR "Man, Modern" OR "Modern Man" OR "Man Taxonomy" OR "Homo sapiens" OR human)) AND (tw:(sports OR sport OR athletics OR athletic OR "Athletic Performance" OR "Athletic Performances" OR "Performance, Athletic" OR "Performances, Athletic" OR "Sports Performance" OR "Performance, Sports" OR "Performances, Sports" OR "Sports Performances" OR exercise OR exercises OR "Physical Activity" OR "Activities, Physical" OR "Physical Activities" OR "Activity, Physical" OR "Exercise, Physical" OR "Exercises, Physical" OR "Physical Exercise" OR "Physical Exercises" OR "Acute Exercise" OR "Acute Exercises" OR "Exercise, Acute" OR "Exercises, Acute" OR "Exercise, Isometric" OR "Exercises, Isometric" OR "Isometric Exercises" OR "Isometric Exercise" OR "Exercise, Aerobic" OR "Aerobic Exercises" OR "Exercises, Aerobic" OR "Exercise Training" OR "Exercise Trainings" OR "Training, Exercise" OR "Trainings, Exercise" OR "Physical

	Fitness" OR "Fitness, Physical")) AND (tw:("Oxidative Stress" OR "Oxidative Stresses" OR "Stresses, Oxidative" OR "Stress, Oxidative")) AND (tw:(saliva OR salivas)))
**GOOGLE SCHOLAR**<br><br>**N=87**	"saliva" + "exercise" + "oxidative stress" -"in vitro" -"systematic review" -book

Table S3. Articles excluded after reading in full and reasons for exclusions

Reference	Reason for exclusion
Caramez F. Respostas adaptativas ao treinamento físico em parâmetros bioquímicos salivares de atletas de futebol: Universidade do Estado do Rio de Janeiro; 2016.	Oxidative stress in blood
Grushin A, Dudnik E, Glazachev O, et al. Assessment of the oxidative-antioxidant status and aerobic performance of elite skiers-racers in the dynamics of training cycle at moderate altitude. 2019;4:12..	Oxidative stress in blood
Lee Y-H, Kim JH, Zhou H, Kim BW, Wong DT. Salivary transcriptomic biomarkers for detection of ovarian cancer: for serous papillary adenocarcinoma. Journal of Molecular Medicine 2012;90:427-434.	Oxidative stress in blood
Puspaningtyas DE, Afriani Y, Mahfida SL, Kushartanti W, Farmawati A. Effect of exercise on lipid peroxidation in student soccer players. Journal of the Medical Sciences (Berkala Ilmu Kedokteran); Vol 50, No 1 (2018)DO - 1019106/JMedSci005001201811 2018.	Oxidative stress in blood
Škrgat S, Marčun R, Kern I, et al. Systemic and airway oxidative stress in competitive swimmers. Respiratory Medicine 2018;137:129-133.	Oxidative stress in blood
Sone R, Matsuba K, Tahara R, et al. Assessment of Salivary Nitric Oxide Levels in Elite University Athletes in Japan: Findings From a Cross Sectional Study Design. J Clin Med Res 2019;11:114-120.	Exercise intervention was not performed
West DJ, Cunningham DJ, Finn CV, et al. The metabolic, hormonal, biochemical, and neuromuscular function responses to a backward sled drag training session. Journal of strength and conditioning research 2014;28:265-272.	No oxidative stress evaluation

Table S4. Quality Assessment Tool for Before-After (Pre-Post) Studies with No Control Group.

Criteria	Biagini, 2020	Damirchi, 2015	Faelli, 2020	Filaire. 2010	Massart, 2012	Menezes, 2019	Nobari, 2021	Ovchinnikov, 2019	Sari-Sarraf, 2016	Sone, 2019	Souza, 2019	Viana-Gomes, 2018
1. Was the study question or objective clearly stated?	Yes	Yes	Yes	Yes	Yes	Yes	Yes	Yes	Yes	No	Yes	Yes
2. Were eligibility/selection criteria for the study population prespecified and clearly described?	No	Yes	Yes	Yes	Yes	Yes	Yes	Yes	Yes	Yes	Yes	Yes
3. Were the participants in the study representative of those who would be eligible for the test/service/intervention in the general or clinical population of interest?	No	No	No	Yes	Yes	No	No	No	Yes	No	No	Yes
4. Were all eligible participants that met the prespecified entry criteria enrolled?	NR	Yes	Yes	Yes	Yes	Yes	Yes	Yes	Yes	Yes	Yes	Yes
5. Was the sample size sufficiently large to provide confidence in the findings?	No	No	Yes	No	No	No	No	No	No	No	No	Yes
6. Was the test/service/intervention clearly described and delivered consistently across the study population?	Yes	Yes	Yes	Yes	Yes	Yes	Yes	Yes	Yes	Yes	Yes	Yes
7. Were the outcome measures prespecified, clearly defined, valid, reliable, and assessed consistently across all study participants?	Yes	Yes	Yes	Yes	Yes	Yes	Yes	Yes	Yes	Yes	Yes	Yes
8. Were the people assessing the outcomes blinded to the participants' exposures/interventions?	NR	Yes	NR	Yes	NR	NR	NR	NR	NR	NR	NR	Yes
9. Was the loss to follow-up after baseline 20% or less? Were those lost to follow-up accounted for in the analysis?	Yes	Yes	NR	Yes	Yes	NR	Yes	Yes	NR	Yes	Yes	Yes
10. Did the statistical methods examine changes in outcome measures from before to after the intervention? Were statistical tests done that provided p values	Yes	Yes	Yes	Yes	Yes	Yes	Yes	Yes	Yes	Yes	Yes	Yes

for the pre-to-post changes?												
11. Were outcome measures of interest taken multiple times before the intervention and multiple times after the intervention (i.e., did they use an interrupted time-series design)?	No	No	No	No	No	No	No	No	No	No	No	Yes
12. If the intervention was conducted at a group level (e.g., a whole hospital, a community, etc.) did the statistical analysis take into account the use of individual-level data to determine effects at the group level?	NA	NA	NA	NA	NA	NA	NA	NA	NA	NA	No	NA
Quality Rating	POOR	GOOD	GOO D	GOOD	GOOD	GOOD	GOOD	GOOD	POOR	POOR	GOOD	GOOD

**Criteria**	**Cavas, 2005**	**Gonzalez, 2008**	**Deminice, 2010**	**Mahdivand, 2013**	**Sariri, 2013**	**Podrigalo, 2015**	**Sant'anna, 2016**	**Kontorschikova, 2017**	**De Araújo, 2018**	**Volodchenko, 2019**
1. Was the study question or objective clearly stated?	Yes	Yes	Yes	No	Yes	Yes	Yes	Yes	Yes	Yes
2. Were eligibility/selection criteria for the study population prespecified and clearly described?	No	No	Yes	Yes	Yes	No	Yes	Yes	Yes	Yes
3. Were the participants in the study representative of those who would be eligible for the test/service/intervention in the general or clinical population of interest?	No	No	No	No	No	No	No	Yes	No	No
4. Were all eligible participants that met the prespecified entry criteria enrolled?	NR	NR	Yes	Yes	Yes	NR	NR	Yes	Yes	Yes
5. Was the sample size sufficiently large to provide confidence in the findings?	No	No	No	No	No	No	No	Yes	No	No
6. Was the test/service/intervention clearly described and delivered consistently across the study population?	No	No	Yes	Yes	Yes	No	Yes	Yes	Yes	Yes

7. Were the outcome measures prespecified, clearly defined, valid, reliable, and assessed consistently across all study participants?	Yes	No	Yes	No	Yes	Yes	Yes	Yes	Yes	No
8. Were the people assessing the outcomes blinded to the participants' exposures/interventions?	NR	NR	NR	NR	Yes	NR	NR	NR	NR	NR
9. Was the loss to follow-up after baseline 20% or less? Were those lost to follow-up accounted for in the analysis?	NR	NR	NR	NR	Yes	Yes	NR	NR	NR	NR
10. Did the statistical methods examine changes in outcome measures from before to after the intervention? Were statistical tests done that provided p values for the pre-to-post changes?	Yes	No	Yes	Yes	Yes	Yes	Yes	Yes	Yes	Yes
11. Were outcome measures of interest taken multiple times before the intervention and multiple times after the intervention (i.e., did they use an interrupted time-series design)?	No	No	No	No	No	No	No	No	No	No
12. If the intervention was conducted at a group level (e.g., a whole hospital, a community, etc.) did the statistical analysis take into account the use of individual-level data to determine effects at the group level?	NA	NA	NA	NA	NA	NA	NA	NA	NA	NA
Quality Rating	FAIR	POOR	GOOD	FAIR	FAIR	FAIR	FAIR	FAIR	GOOD	FAIR
